# Deep neural networks for inferring binding sites of RNA-binding proteins by using distributed representations of RNA primary sequence and secondary structure

**DOI:** 10.1186/s12864-020-07239-w

**Published:** 2020-12-17

**Authors:** Lei Deng, Youzhi Liu, Yechuan Shi, Wenhao Zhang, Chun Yang, Hui Liu

**Affiliations:** 1 School of Computer Science and Engineering, Central South University, Changsha, 410075 China; 2 Aliyun School of Big Data, Changzhou University, Changzhou, 213164 China; 3 Department of Obstetrics, The Affiliated Changzhou No.2 People’s Hospital of Nanjing Medical University, Changzhou, China

**Keywords:** RNA-binding proteins, Binding sites, Distributed representation, k-mer, Deep learning, Convolutional neural network, Bidirectional long short term memory network

## Abstract

**Background:**

RNA binding proteins (RBPs) play a vital role in post-transcriptional processes in all eukaryotes, such as splicing regulation, mRNA transport, and modulation of mRNA translation and decay. The identification of RBP binding sites is a crucial step in understanding the biological mechanism of post-transcriptional gene regulation. However, the determination of RBP binding sites on a large scale is a challenging task due to high cost of biochemical assays. Quite a number of studies have exploited machine learning methods to predict binding sites. Especially, deep learning is increasingly used in the bioinformatics field by virtue of its ability to learn generalized representations from DNA and protein sequences.

**Results:**

In this paper, we implemented a novel deep neural network model, DeepRKE, which combines primary RNA sequence and secondary structure information to effectively predict RBP binding sites. Specifically, we used word embedding algorithm to extract features of RNA sequences and secondary structures, i.e., distributed representation of k-mers sequence rather than traditional one-hot encoding. The distributed representations are taken as input of convolutional neural networks (CNN) and bidirectional long-term short-term memory networks (BiLSTM) to identify RBP binding sites. Our results show that deepRKE outperforms existing counterpart methods on two large-scale benchmark datasets.

**Conclusions:**

Our extensive experimental results show that DeepRKE is an efficacious tool for predicting RBP binding sites. The distributed representations of RNA sequences and secondary structures can effectively detect the latent relationship and similarity between k-mers, and thus improve the predictive performance. The source code of DeepRKE is available at https://github.com/youzhiliu/DeepRKE/.

**Supplementary Information:**

The online version contains supplementary material available at (doi:10.1186/s12864-020-07239-w).

## Background

RNA-binding proteins (RBPs) have important functions in many biological processes, e.g. gene regulation, RNA editing, mRNA processing and other biological processes [1, 2]. It is estimated that 6% -8% of the proteins in eukaryotes are RNA binding proteins, but so far only a few RNA binding proteins (HuR, AUF1, TTP, TIA1, CUBBP2, etc) have been approved to be specifically involved in mRNA stability, translation and other levels of gene regulation [3–7]. Therefore, the identification of RBP binding sites is crucial to understanding the mechanism of biological processes. Recently, various of high-throughput biochemical methods have been proposed to study and analyze the proteins-RNA complexes to identify the binding sites of RNA molecules, among which the popular ones are CLIP-Seq [8], RNACompete [9], eCLIP [10] and PAR-CLIP [11]. However, these techniques still cost-heavy and time-intensive, which hampers the exploration of RBP binding sites.

With the increasing volume of verified RBP binding sites, quite a few studies focused on developing computational prediction models based on the known RBP binding sites. As is shown in Table 1, these methods mostly employ RNA sequence and structural information to predict protein-RNA interactions. For instance, RNAcontext [12] uses position weight matrix (PWM) of RNA sequence and secondary structure profile to predict the binding preference of RBP. RCK [13] is an extension of RNAcontext, which uses a novel k-mer-based model to further improve the predictive performance. GraphProt [14] adopts the form of graph coding to integrate RNA sequence and structure into the graph kernel to generate a feature vector of more than 3,000 dimensions, which is subsequently used as the input of an SVM model to predict RBP binding preference. IONMF [15] proposes a feature representation method of orthogonal matrix eigendecomposition, which integrates the k-mer sequence, secondary structure, gene ontology (GO) information and region type as input into a machine learning model to predict binding sites. Oli [16] uses k-mer frequency as input feature into an SVM classifier to predict RNA-protein interactions. Rather than commonly constructing a binary classification task, RNAcommender [17] adopts recommendation system to prioritize RNA against undeveloped RNA binding proteins by disseminating available interaction information based on protein domain composition and RNA predicted secondary structure.

**Table 1:** Computational methods for RBP binding preference prediction

Method	Sequence	Structure	Reference
RNAcontext	Yes	Yes	[12]
GraphProt	Yes	Yes	[14]
iONMF	Yes	Yes	[15]
Oli	Yes	Yes	[16]
RNAcommender	Yes	Yes	[17]
RCK	Yes	Yes	[13]
DeepBind	Yes	No	[18]
Deepnet-rbp	Yes	No	[19]
DanQ	Yes	No	[20]
iDeepS	Yes	Yes	[21]
iDeepV	Yes	No	[22]
Pysster	Yes	Yes	[23]
DLPRB	Yes	Yes	[24]

“Yes” and “No” means whether the computational methods uses sequence and structure information to predict the binding site

Recently, deep learning [25] has achieved remarkable success in many fields, such as image processing [26], natural language processing [27] and speech recognition [28], and thus drawn greater attention from bioinformaticians [29]. Through multiple hidden layers that perform feature transformation in the deep neural network, the feature representation in the original space is mapped into a new feature space, which makes the task of interest smoother. Based on the set of verified RBP binding sites, more and more studies use deep learning to predict RBP binding sites. When constructing the input of the neural network, most methods adopt one-hot coding, e.g., the four nucleotides A, C, G, and T are encoded as (0, 0, 0, 1), (0, 1, 0, 0), (0, 0, 1, 0) and (0, 0, 0, 1), and thus a RNA sequence of length *L* is transformed into a matrix of size 4*L. DeepBind [18] is the first to use CNN (Convolutional Neural Network) [30] to predict protein-DNA/RNA binding preferences based on one-hot coding of nucleotide sequences. DanQ [20] and iDeepS [21] also adopt one-hot encoding of nucleotide sequences as input of deep learning models to predict protein-RNA binding preferences. Of note, iDeepS additionally makes use of the information of RNA secondary structure profiles. Pysster [23] exploits a novel strategy to expand the one-hot coding to predict protein-RNA binding preferences. It combines RNA sequence and secondary structure alphabet into an extended alphabet composed of arbitrary characters, which integrates RNA Sequence and structure input strings. Then, the proposed string is encoded as one-hot and used as the input. However, a fatal disadvantage of traditional one-hot coding is the problem of curse of dimensionality. Accordingly, deep-rbps [19] uses k-mer frequency coding to encode sequence, secondary structure and tertiary structure into a unified feature representation, which is subsequently fed into a multi-modal DBN [31] to predict RBP binding sites and motifs. In general, k-mer frequency coding greatly reduces the dimension of the input of deep neural network. Taking 4-mer peptide for example, in all possible cases, only a total of 256 kinds of 4-mer (AAAA, AAAC... TTTT) peptide are required to be counted the frequencies included in the sequence, resulting to a 256-D final vector. While k-mer frequency coding can effectively reduce the dimension, it ignores the position information of the sequence that is actually important for the prediction of RBP sites. Moreover, k-mer frequency coding does not consider contextual correlation. Inspired by the field of natural language processing, DNA2vec [32], BioVec [33], seq2vec [34], and Gene2Vec [35] heuristically use word2vec [36] to obtain a distributed representation from genomic sequences without supervision. iDeepV [22] applies the word embedding method to learn distributed representation of k-mers, and greatly improves the prediction accuracy.

In this paper, we present a novel deep neural network, DeepRKE, which consists of CNN and bidirectional LSTM, to infer latent RBP binding sites. We used the word embedding model to build distributed representations of RNA sequences and secondary structures, and input them into a deep neural network to predict RBP binding sites. The skip-gram algorithm [37] uses the input of current word to predict the surrounding context words, and can effectively capture the contextual information. Thereby, we used skip-gram algorithm to learn a k-dimensional distributed representation of RNA sequence and RNA secondary sequence in a low-dimensional space. DeepRKE has the following contributions: 1) k-mer embedding is used to represent both RNA sequence and RNA secondary structure instead of traditional one-hot encoding. 2) We use three CNN modules, two modules are used to extract the features of RNA sequence and secondary structure respectively, and the third module is used to capture the relationship between sequence and structure. 3) DeepRKE can handle the sequences with variable length. We evaluated DeepRKE on two large-scale benchmark datasets, and also assessed its performance on sequences with fixed length and variable length. The experimental results demonstrated that deepRKE achieved better performance than five competitive methods.

## Results

In this part, First we evaluated DeepRKE with other five state-of-the-art methods on two large-scale benchmark datasets RBP-24 [13] and RBP-31 [38]. Next, We deleted the secondary structure profile from the input, and then only used the sequence as the input of DeepRKE to judge the impact of RNA secondary structure on the prediction ability from the final prediction effect. In addition, we also evaluated the performance improvement of Deep- RKE by using distributed representation or not, as well as one-hot encoding. Finally, for more insights into the performance improvement by BLSTM, we compare DeepRKE with a variant using only CNNs without BLSTM layer.

### DeepRKE learning framework

**Fig. 1:**
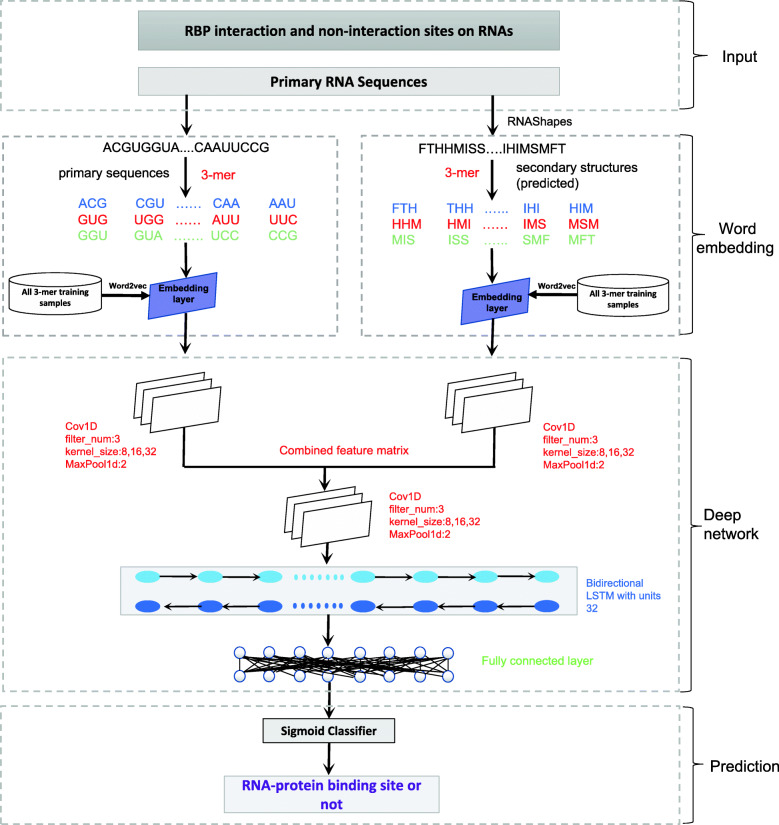
The illustrative flowchart of DeepRKE learning framework. First, we use RNAShapes to predict the RNA secondary structure from primary sequences. Second, word embedding algorithm is used to learn the distributed representations of 3-mers from primary sequences and secondary structures. Third, the learned distributed representations are fed into two CNNs (one is for RNA sequence and the other for secondary structures) to transform sequence and structure features, which are in turn input into a CNN module and a bidirectional LSTM layer followed by two fully connected layer. Finally, we use a sigmoid classifier to predict the probability of being RBP binding sites

We implemented the learning framework of DeepRKE to infer RNA-proteins binding site on RNAs, as shown in Fig. 1. First, we used RNAShapes [39] to predict the RNA secondary structure. Second, we used the word2vec algorithm to learn distributed representation of 3-mers of RNA sequences and secondary structure sequences. The distributed representations were used as the input of two CNNs (one is for RNA sequence and the other is for secondary structure) to transform features of sequences and structure, respectively. Next, we combined the output features and fed them into another CNN, which was followed by a bidirectional LSTM and two fully connected layers. Finally, a sigmoid function was used to predict the probability of RBP binding sites.

### Performance comparison to counterpart methods

We evaluated DeepRKE with other multiple existing methods on two large-scale benchmark datasets, including RBP-24 [14] and RBP-31 [40]. The RBP-24 dataset contains RNA sequences with variable length ranging from 150 to 375, while the RBP-31 dataset contains fixed-length RNA sequences with 101 nucleotides. The counterpart methods are GraphProt, deepnet-rbp, DeepBind, iDeepS and iDeepV. Note that iDeepV and DeepBind use only sequences, while DeepRKE, iDeepS and GraphProt use both sequences and secondary structures. DeepBind and iDeepS use one-hot encoding to represent RNA sequence, DeepRKE and iDeepV use distributed representations of RNA sequence.

First, we conducted the performance evaluation on the RBP-24 dataset with GraphProt, deepnet-rbp, DeepBind and iDeepV that can handle variable sequences. Table 2 shows the number of training and test samples gained by each biochemical assay included in the RBP-24 dataset, as well as the corresponding AUC performance measures (detailed results are shown in Additional file 1). It can be found that among the 24 RBPs, the average AUC of DeepRKE is 0.934, higher than that of the secondary method DeepBind which is 0.917. GraphProt obtained the worst performance 0.887. More importantly, DeepRKE obtains the best AUCs on 18 out of total 24 sets of RBPs. It is worth noting that the performance of the four deep learning-based methods perform far more superior to the GraphProt that is traditional machine learning-based. iDeepV adopts the distributed representation, but merely uses RNA sequences. As a result, its performance is better than GraphProt and deepnet-rbp, but worse than DeepRKE. For example, on ALKBH5 and C17ORF85, DeepRKE obtains the AUC values of 0.740 and 0.824, which is an increase of 15% and 11% compared to the AUCs of 0.643 and 0.74 achieved by iDeepV, respectively. For some RBPs, DeepRKE greatly boost the prediction performance, e.g. DeepRKE increases the AUC values by 18% and 14% compared to GraphProt on Ago2 and TIAL1.

**Table 2:** Performance comparison between DeepRKE, GraphProt, deepnet-rbp, DeepBind and iDeepV on RBP-24 dataset

RBP	#positives	#negatives	GraphProt	deepnet-rbp	DeepBind	iDeepV	DeepRKE
ALKBH5 PAR-CLIP	1213	1197	0.680	0.714	0.668	0.643	**0.740**
C17ORF85 PAR-CLIP	1860	1849	0.800	0.820	0.755	0.740	**0.824**
C22ORF28 PAR-CLIP	9369	9136	0.751	0.792	0.809	0.823	**0.832**
CAPRIN1 PAR-CLIP	8140	7901	0.855	0.834	**0.888**	0.824	0.869
Ago2 HITS-CLIP	48,095	44,251	0.765	0.809	0.879	0.886	**0.900**
ELAVL1 HITS-CLIP	8595	8436	0.955	0.966	**0.980**	0.966	0.978
SFRS1 HITS-CLIP	19,438	17,195	0.898	0.931	0.929	0.905	**0.945**
HNRNPC iCLIP	21,472	19,794	0.952	0.962	**0.979**	**0.979**	0.978
TDP43 iCLIP	92,031	75,079	0.874	0.876	0.930	0.935	**0.954**
TIA1 iCLIP	18,049	16,135	0.861	0.891	0.929	0.941	**0.942**
TIAL1 iCLIP	42,332	36,652	0.833	0.870	0.922	0.929	**0.946**
Ago1-4 PAR-CLIP	36,902	31,310	0.895	0.881	0.919	0.925	**0.932**
ELAVL1 PAR-CLIP(B)	9464	9283	0.935	0.961	0.961	0.962	**0.980**
ELAVL1 PAR-CLIP (A)	27,275	23,974	0.959	0.966	0.972	0.973	**0.978**
EWSR1 PAR-CLIP	16,292	14,720	0.935	0.966	0.969	0.962	**0.971**
FUS PAR-CLIP	34,581	31,480	0.968	0.980	0.983	0.976	**0.988**
ELAVL1 PAR-CLIP(C)	125,202	113,686	0.991	0.994	0.989	0.990	**0.996**
IGF2BP1-3 PAR-CLIP	8539	6838	0.889	0.879	0.939	0.923	**0.943**
MOV10 PAR-CLIP	13,793	12,987	0.863	0.854	0.899	0.896	**0.920**
PUM2 PAR-CLIP	9116	8227	0.954	**0.971**	0.964	0.965	0.965
QKI PAR-CLIP	10,276	9142	0.957	**0.983**	0.973	0.965	0.975
TAF15 PAR-CLIP	7298	6606	0.970	0.983	0.978	0.978	**0.985**
PTB HITS-CLIP	44,574	43,700	0.937	**0.983**	0.944	0.936	0.953
ZC3H7B PAR-CLIP	20,962	20,018	0.820	0.796	0.875	0.883	0.914
Mean AUC			0.887	0.902	0.917	0.913	0.934

Note: boldface is the best experimental results for this experiment

**Fig. 2:**
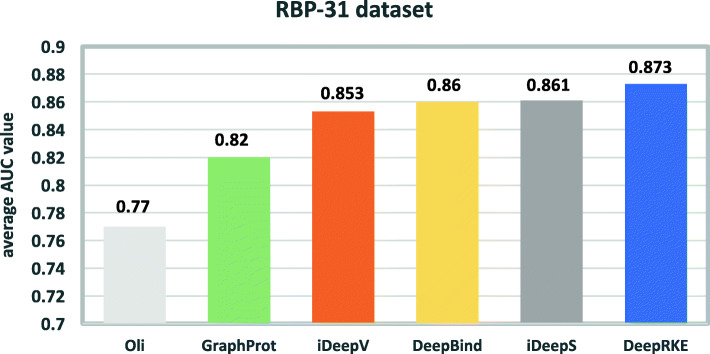
Performance comparison between DeepRKE, iDeepV, iDeepS, DeepBind and GraphProt on RBP-31 dataset. All methods are run on the same training and independent test set across 31 set of RBPs (x-axis)

On RBP-31 dataset, we also compared DeepRKE to iDeepS and Oli’s method [16]. The AUC values of each method are shown in Additional file 2. The average AUCs of six competitive methods on 31 set of RBPs are illustrated in Fig. 2, where the performance of DeepRKE is significantly superior to other 5 methods with the average AUC of 0.873.

Note that the current state-of-the-art method iDeepS gets an average AUC of 0.86, which is still slightly worse than DeepRKE. In fact, our method performs better than iDeepS on quite a few sets of RBPs. For example, DeepRKE increases the AUC by 8.2%, 5.1%, 5%, 11%, 7.5% on the Ago/EIF, Aog2-MNase, Ago2-1, IGFBP1-3 and MOV10, respectively. Moreover, we compared DeepRKE with GraphProt, which is a structure profile-based method and demonstrates better performance than RNAcontext [12]. GraphProt has the average AUC of 0.82, which is inferior to 0.873 of DeepRKE. Remarkably, DeepRKE achieves better AUCs than GraphProt in all experiments. The average AUC of Oli is 0.77, which is significantly lower than DeepRKE. Oli even obtains the performance close to random guessing on some sets of RBPs, e.g. on Ago2-MNase its AUC is only 0.512.

In summary, DeepRKE achieves significant performance on both RBP-24 and RBP-31 datasets. DeepRKE not only outperforms the current methods for predicting RBP binding sites, but also successfully handles input sequences with variable length, which fails to be addressed by iDeepV and iDeepS.

### RNA secondary structure impacts predictive power

The results in Table 2 has somewhat demonstrated the importance of RNA secondary structure, e.g., the methods that adopts the secondary structure information commonly outperform those use only sequence information. The proposed deep learning framework allows us to more strictly investigate the impact of RNA structure on performance, namely, whether and what extend the secondary structure can contribute to the prediction of RBP binding site. Accordingly, we removed the secondary structural profiles from input, and then took only the sequence as the input of our model. For clarity, we referred to this simplified model as DeepRKE-, indicating that RNA structure information is not taken into account.

**Fig. 3:**
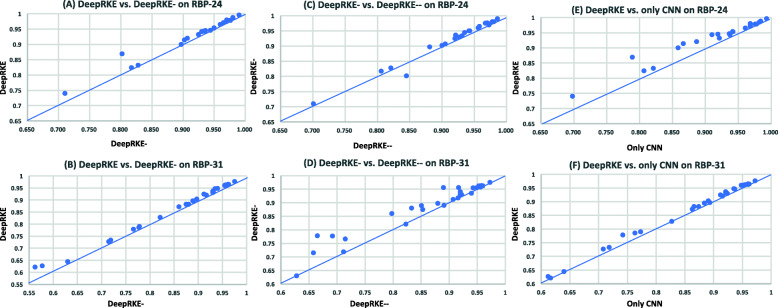
Performance comparison of the models with or without distributed representation of sequences and secondary structural profiles. The performance was evaluated in terms of AUROC on RBP-24 and RBP-31 dataset. DeepRKE is our proposed model, DeepRKE- model is without RNA secondary structure, and DeepRKE- - is without RNA secondary structure and distributed representation of sequence, using one-hot encoding instead. **a**-**b** Performance comparison between DeepRKE and DeepRKE- on two datasets. **c**-**d** Performance comparison between DeepRKE- and DeepRKE- - on two datasets. **e**-**f** Performance comparison between models with only CNN laryer and CNN+BiLSTM layer on two datasets

As shown in Table 1 and Fig. 3a, DeepRKE- achieves an average AUC of 0.924 on RBP-24 dataset, which is lower that of DeepRKE 0.934. We observed significant decrease in performance (with AUC score reduced by >1%) in eight RBPs, i.e. ALKBH5, C17ORF85, CAPRIN1, SFRS1, TIA1, ELAVL1(B), MOV10, ZC3H7B. Especially, on the two proteins ALKBH5 and CAPRIN1, AUC decreases by 3% and 6%, respectively. As shown in Fig. 3b, on RBP-31 dataset, DeepRKE- achieves an average AUC of 0.863 over 31 sets of RBPs, which is still worse than that of 0.873 achieved by DeepRKE. On the two proteins Ago2-MNase and ELAVL1-MNase, AUC decreases by 6% and 5%, respectively. The result implies that RNA secondary structure significantly contributes to the prediction of RBP binding sites.

### Distributed representation significantly improves performance

Rather than traditional one-hot encoding, DeepRKE adopts distributed representations of RNA sequence to capture the high-order dependencies among nucleotides, leading to dimension-reduced feature vectors. To justify the advantage of distributed representation in identifying binding site of RNA-binding proteins, we removed the secondary structural profiles (as it can not be represented by one-hot encoding) and took one-hot encoding of RNA sequence as the input of deep learning framework. For convenience, we referred to this model as DeepRKE- -, indicating that both RNA secondary structure and distributed representations of RNA sequence are excluded when constructing the input.

**Figure** 3c-d shows the performance comparison between DeepRKE- and DeepRKE- - on dataset RBP-24 and RBP-31. We can see that the performance of DeepRKE- is better than DeepRKE- - on RBP-24 dataset, except for the CAPRIN1 protein. Specifically, DeepRKE- achieves an average AUC of 0.863, which is better than 0.841 of DeepRKE- - on RBP-31 dataset. DeepRKE- performs significantly better than DeepRKE- - on some proteins, e.g. Ago/EIF, Ago2-2, eIF4AIII-1, hnRNPL-2. The results suggest that the distributed representations of RNA sequence can significantly improve the performance for identifying binding site of RNA-binding proteins.

### BiLSTM helps to improve prediction accuracy

In our proposed learning framework, BiLSTM is used to identify long-term dependent information of the extracted sequence and secondary structure feature [38]. For further evaluation of the performance enhancement by BiLSTM, we compared DeepRKE with a variant model using only CNN models that removes the BiLSTM layer. For the sake of fairness, we used the same parameters and architecture for CNN layer, including filter size, kernel size, learning rate and maxpool1d size. The results are shown in Fig. 3e-f, where DeepRKE achieves better performance than the CNN-only model on the two datasets. In particular, DeepRKE achieves an average AUC of 0.934 on RBP-24 dataset, which is better than that of 0.916 achieved by the CNN-only variant. Similarly, DeepRKE achieves an average AUC of 0.873 on RBP-31 dataset, which is still better than an average AUC of 0.863 of the CNN-only variant. The results suggest BiLSTM can effectively improve the performance of predicting protein-RNA binding sites.

## Discussion

In this paper, we demonstrated that distributed representation can significantly improve the predictive ability in modeling protein-RNA binding. In fact, we can also obtain the distributed representations by using UTR sequences, and thus our method is not limited to the application of RBP-24 and RBP-31 benchmark datasets.

With the incorporation of biLTSM layer and secondary structure information, our method outperforms all counterpart methods in predicting protein-RNA binding sites. However, we can make advantage of RNA tertiary structure or region type to further improve the performance, similar to deepen-rbp [19], iDeep [41]. Compared to the CNN-only model, we also confirmed the advantage of recurrent neural networks in capturing high-order interdependence of sequences and secondary structures. In addition, we can also take the correlation between RBPs into consideration in the construction of our model, as done in protein-lncRNA interactions prediction [42] and protein-protein interactions prediction [43].

Note that only a small fraction of RNA sequences have been detected by CLIP-seq assays, the vast majority of sequences are not found so far. Therefore, the number of negative samples is much higher than that of positive samples in the real world. However, we built balanced training sets in this study, for that imbalanced datasets often lead to biased machine learning models with preference on overwhelming class (e.g. most of sequences are classified to negative samples), which makes trained models useless. More verified RBP binding sites are expected to improve the predictive power of computational models.

Although DeepRKE achieves state-of-the-art performance, it can not identify the binding motifs directly from learned convolve filters. We plan to extend DeepRKE to identify the binding motifs in our future work. For example, we can use the DeepRKE model to assign binding potential scores to all sequences of interest, and then select high-confidence candidates to extract the binding motifs.

## Conclusion

In this paper, we present a novel deep neural network model, DeepRKE, to predict RBP binding sites. DeepRKE combines the primary RNA sequence and secondary structure into a unified learning framework. The novelty of DeepRKE lies in that we use the skip-gram model in word2vec to learn the distributed representation of RNA sequences and RNA structure. Also, we introduced the BiLSTM layer into the deep learning model to capture the high-order interdependence of sequences and secondary structures. We evaluated DeepRKE on two RBP binding benchmark sets derived from the CLIP-seq, and the results demonstrated that DeepRKE achieves better AUCs than other competitive methods. Our results suggest that distributed representation of k-mers sequence helps to improve the prediction performance for identifying the binding sites of RNA-binding protein. The BiLSTM layer also contributes significantly to the enhancement of predictive ability.

## Methods

### Datasets

Two large-scale datasets derived from human CLIP-seq: RBP-24 and RBP-31 are used as the benchmark datasets. The detail of the datasets are as below:

1. RBP-24 dataset is used by GraphProt [14] as the training and test set. It consists of 24 experiments covering 21 RBPs, and the RNA sequences are variable length ranging from 150 to 375. For each experiment, the positive sites are downloaded from do-RiNA [40], the negative sites are created by shuffling the coordinates of binding sites within all genes with at least one binding site using bedtools shuffle [44]. We further randomly select a third of the original training data as validation set, and the remaining two-thirds as training set, and the independent testing set is the same test set used in GraphProt. The numbers of training positives and negatives of each experiment are listed in Table 2.
2. RBP-31 dataset includes training and test samples with fixed-length RNA sequences of 101 nucleotides collected in iONMF [15], which can be downloaded from https://github.com/mstrazar/ionmf. In this dataset, the CLIP-seq data consists of 19 proteins with 31 experiments, and their annotations are based on human assembly hg19. As described in iONMF, each nucleotide within clusters of interaction sites derived from CLIP-seq is considered as binding sites. To reduce redundancy, the positive binding sites with the highest cDNA count and without consecutive sites on genome are further randomly selected. Finally, among those sites with less than 15 nucleotides apart, one site with the highest cDNA counts was selected as the positive sample. The negative sites were sampled from genes that were not identified as an interacting partner in any of 31 experiments. As a result, 4,000 cross-linked sites are used for training, 1,000 samples for validation, and other 1,000 samples for independent test.

In addition, as DeepRKE requires RNA secondary structural sequence as input, we fed RNA sequence into RNAShapes [39] to obtain the dot parenthesis diagram, which is subsequently used as the input of EDeN to obtain the RNA secondary structure sequence. The RNAshapes have six generic shapes: stems (S), multiloops (M), hairpins (H), internal loops (I), dangling end (T) and dangling start (F).

### Distributed representation of k-mer sequences

Word2Vec is a model proposed for learning semantic knowledge from a large number of textual corpora in an unsupervised manner, which is widely used in natural language processing. We extend the usage of word2vec to obtain the distribution representation of k-mer sequences. Based on the theory of distribution hypothesis, distributed representation is a strategy to obtain the semantic representation of words by using the symbiotic matrix. Word embedding is a multi-dimensional vector of real value which is mapped from the vocabulary words or phrases. It can capture the potential relationship between context and the target word. Generally, Word2Vec provides two architectural options: CBOW and skip-gram. CBOW can predict the current word based on the surrounding context, while the skip-gram uses the current word to predict the surrounding context. In this paper, we use skip-gram to learn the distributed representation of k-mers.

Given word sequence *s*_1_,*s*_2_,*s*_3_.....*s*_*k*_, skip-gram learns the word representations using the co-occurrence information of words within a context window. It maximizes the following objective function:

1 $$ \frac{1}{k}\sum\limits^{k}_{i=1}\sum\limits_{-m\leq j\leq m, j\neq0}logp\left(S_{t+j}\mid S_{j}\right)  $$

where *m* is the context window size, and the conditional probability *p* is defined as follows:

2 $$ p\left(S_{o}\mid S_{c}\right)=\frac{exp\left({v'}_{s_{o}}^{T}v_{s_{c}}\right)}{{\sum\nolimits}_{s=1}^{V}exp\left({v'}_{s}^{T}v_{s_{c}}\right)}  $$

where *V* is the size of vocabulary, $v_{s_{c}}$ is word vector of the center word, v _*s*_ and v$_{s}^{'}$ is the input and output vector representation of word *s*, respectively.

Because of the computational infeasibility, the *logp*(*S*_0_∣*S*_*i*_) is approximated using negative sampling:

3 $$ log\sigma\left({v'}_{s_{o}}^{T}v_{s_{c}}\right)+\sum\limits^{k}_{i=1}E_{s_{i}\sim P_{a}(s)}\left[log\sigma\left(-{v'}_{s_{i}}^{T}v_{s_{c}}\right)\right]  $$

where *σ*=1/(1+*exp*(−*x*)).

Recently, BioVec [34], seq2vec [33], dna2vec [32] and Gene2Vec [35] have also successively applied Word2Vec to encode biological sequences, and so did we. Specifically, we consider each k-mer as a word, each sequence as a sentence, all dataset sequences as corpus, as a result, we can learn the distributed representation of k-mers by using skip-gram algorithm. We split all sequences into 3-mer form. For example, the sequence AUUGC has 5 bp and its secondary structure is FHSIH, we can convert it to AUU,UUG,UGC,FHS,HSI,SIH. Based on the 3-mers derived from all training sequences, we trained the skip-gram algorithm using negative sampling, to predict the context of the targeted k-mer. Finally, we obtained the distributed representation for 4^3^=64 (sequences) and 5^3^=125 (structures) with 3-mers.

### Learning framework of DeepRKE

**Convolutional neural network:** In our method, we employ three CNNs as feature extractors [45], which take as input the distribution representation of k-mer derived from RNA sequences and structures. The convolutional layer learns the weight parameters of the convolution filters, and outputs the matrix inner product between the input matrix and filters. After convolution, a rectified linear unit (ReLU) is applied to sparsify the output of the convolution layer and keep only positive matches to avoid the vanishing gradient problem [46]. Finally, a max pooling operation is used to reduce the dimensionality and yield invariance to small sequence shifts by pooling adjacent positions within a small window. DeepRKE includes three CNN modules, two of which are used to extract the features of RNA sequences and structures, respectively, and the third is designed to extract the high-order features between sequences and structures. The convolution kernel size of each CNN module is 8,16,32, and maxpooling size is 2.

**Long Short Term Memory networks:** In order to further detect long-term interdependence of sequence and secondary structure, we introduced a bidirectional long and short-term memory network (BiLSTM) [47] into our model. Compared to traditional recurrent neural networks (RNNs), LSTM has advantages in addressing the gradient vanishing or exploding, as well as capturing long-term dependency. Especially, BiLSTM exploits the contextual information on both sides.

As LSTM iterates over each element of input, it firstly determines what information the forgetting gate layer should exclude based on previous input. The input gate layer is then used to determine what information should be stored for the next layer and update the current state value. Finally, the output gate layer determines which parts of the state value should be output. Taking a sequence {*x*}$_{t=1}^{T}$ as input, the LSTM have the hidden states {*h*}$_{t=1}^{T}$, cell states {*C*}$_{t=1}^{T}$, and it outputs a sequence {*o*}$_{t=1}^{T}$. The above steps can be formulated as follows:

4 $$ \begin{aligned} & f_{t}=\sigma\left(W_{f}x_{t}+U_{f}h_{t-1}+b_{f}\right),\\ & i_{t}=\sigma\left(W_{i}x_{t}+U_{i}h_{t-1}+b_{i}\right),\\ & c_{t}=f_{t}\odot c_{t-1}+i_{t}\odot tanh\left(W_{c}x_{t}+U_{c}h_{t-1}+b_{c}\right),\\ & o_{t}=\sigma\left(W_{o}x_{t}+U_{o}h_{t-1}+b_{o}\right),\\ & h_{t}=o_{t}\odot tanh(c_{t}) \end{aligned}  $$

where ⊙ denotes element-wise multiplication, the *σ* is the Logistic Sigmoid function and tanh is the tanh function to force the values to be between -1 and 1. *W*_*f*_,*W*_*i*_,*W*_*o*_,*U*_*f*_,*U*_*i*_ and *U*_*o*_ are the weights and *b*_*f*_,*b*_*i*_,*b*_*c*_ and *b*_*o*_ are the bias.

In DeepRKE, a bidirectional LSTM (BiLSTM) is used to scan from left to right and from right to left, concatenating the output in each direction as a final output for subsequent classification. The number of neurons in this layer was set to 32, thereby the output size is 64.

## Supplementary information

**Additional file 1** The file includes the performance measures of competitive methods on the RBP-24 dataset. The AUC values of using DeepRKE- and without RNA secondary structure sequence, as well as the AUC values using DeepRKE- without RNA secondary structure and distributed representation of sequences.

**Additional file 2** The file includes the performance measures of competitive methods on the RBP-31 dataset. The AUCs of using DeepRKE- and without RNA secondary structure sequence, as well as the AUC values of using DeepRKE- without RNA secondary structure and distributed representation of sequences.

## Data Availability

The source code and data sets of DeepRKE are freely available at https://github.com/youzhiliu/DeepRKE/.

## Abbreviations

CLIP: UV-crosslinked immunoprecipatation
RBP: RNA binding proteins
PWM: Position weight matrix
GO: Gene ontology
CNN: Convolutional neural networks
LSTM: Long-term short-term memory networks
BiLSTM: Bidirectional long-term short-term memory networks
DBN: Deep belif neural network
